# Unraveling life expectancy and death spectrum changes of registered residents (hukou) in Quzhou, China, 2015–2023: a study using Arriaga decomposition method

**DOI:** 10.3389/fpubh.2025.1687798

**Published:** 2025-11-28

**Authors:** Jie Pan, Yewei Huang, Shilin Jiao, Biyun Zheng, Zhiying Yin, Zhijuan Gan

**Affiliations:** 1 Department of NCDs Control and Prevention, Quzhou Center for Disease Control and Prevention, Quzhou, Zhejiang, China; 2 Department of NCDs Control and Prevention, Changshan County Center for Disease Control and Prevention, Quzhou, Zhejiang, China

**Keywords:** life expectancy, cause-eliminated life expectancy, death spectrum, Arriaga decomposition method, loss of life expectancy rate

## Abstract

**Objective:**

In less-developed, rapidly aging areas like Quzhou, in-depth analyses of life expectancy change are scarce. Using Arriaga decomposition method, this study quantify age- and cause-specific contributions to changes in Quzhou’s life expectancy in 2015–2023, assess the major causes of death affect registered residents (hukou), and provide evidence to inform targeted health policies and resource allocation in Quzhou and other similar underdeveloped, aging regions.

**Methods:**

The death data was sourced from the Zhejiang Province Chronic Disease Monitoring Information Management System, while population data was sourced from the Zhejiang Province Public Security Department. Residents in the death and population data refer to individuals registered in the Household Registration (hukou) System in Quzhou. Chiang abridged life table was compiled using Excel 2019. The Arriaga decomposition method was employed to analyze the differences in life expectancy across different periods.

**Results:**

Over the period 2015–2023 in Quzhou, life expectancy increased by 1.55 years. Age groups 55–80 made significant positive contributions, while 0- and 85- groups saw decreased contributions. Malignant neoplasms showed the most significant positive growth in contribution rates, increasing by 57.10%, while respiratory diseases exhibited the most notable negative growth, decreasing by 77.07%. The top 10 causes of death shifted, with malignant neoplasms consistently leading. The loss of life expectancy rate due to malignant neoplasms consistently ranked first at 4.22, 3.89, and 3.32% in 2015, 2019, and 2023, respectively. The loss of life expectancy due to respiratory diseases decreased from 3.22 years in 2015 to 1.92 years in 2023.

**Conclusion:**

The life expectancy from 2015 to 2023 showed an increasing trend, with the Arriaga decomposition method revealing positive contributions of infants and older adults aged 60–85 to the growth in life expectancy. However, adjustments in health strategies are needed to address the negative contributions in the 85- age group. Malignant neoplasms and respiratory system diseases, among other chronic illnesses, showed high contribution rates to the increase in life expectancy, emphasizing the need for a comprehensive system for the prevention and control of chronic diseases.

## 1 Introduction

Life expectancy refers to the average number of years a person is expected to live at a specific age, and it is an important indicator of the health status of a country or region (1, 2). As a core indicator reflecting the socioeconomic development level, medical and health conditions, and environmental factors, life expectancy is widely recognized globally (3–6). In recent years, global life expectancy has shown a general increasing trend. According to data from the World Health Organization, the global average life expectancy reached 73.1 years in 2019, an increase of 6.3 years from 66.8 years in 2000 (7). This growth is attributed to the combined efforts of countries in advancing medical technology, improving public health policies, and promoting socioeconomic development. However, there are still significant differences in life expectancy among different countries and regions, especially between developed and developing countries (8, 9). In China, with rapid economic development and improvements in medical and health standards, life expectancy has significantly increased. According to data from the National Bureau of Statistics, China’s life expectancy reached 78.6 years in 2023. Zhejiang has made significant efforts in improving medical infrastructure, promoting health education, and enhancing disease prevention, providing important guarantees for the increase in life expectancy. Life expectancy has risen from 75.6 years in 2003 to 82.3 years in 2022.

Current research on life expectancy mainly focuses on several aspects: analyzing the historical trends of life expectancy to reveal the underlying socioeconomic factors, studying the impact of different causes of death on life expectancy through decomposition methods, and using statistical models to predict the future trends of life expectancy (3, 10, 11). These studies not only help understand the driving factors behind changes in life expectancy but also provide scientific basis for formulating public health policies and resource allocation.

The Arriaga decomposition method, as an effective analytical tool, has been widely used in life expectancy studies (2, 10, 12, 13). By analyzing the contribution of causes of death to changes in life expectancy, it helps researchers identify key factors influencing population health. In recent years, an increasing number of studies have utilized the Arriaga decomposition method to analyze changes in life expectancy in different regions and population groups, providing important references for the formulation of health intervention measures (2, 11, 12, 14).

Quzhou, situated in western Zhejiang, is relatively less developed economically; according to the Zhejiang Statistical Yearbook (2023), its gross regional product ranks third from the bottom among the province’s 11 prefecture-level cities. Meanwhile, population aging is notable: the share of registered residents aged 60 + is 25.02%, ranking 5th provincially. This dual context—constrained economic resources alongside mid-to-high population aging—may produce age- and cause-specific contribution patterns to life expectancy change that diverge from provincial averages. Existing province-level studies cannot identify which age groups and causes drive the marginal changes in life expectancy in such settings. Therefore, this study uses the Arriaga decomposition method to analyze changes in life expectancy and the death spectrum in Quzhou, China, 2015–2023. By evaluating the impact of different causes of death on life expectancy, this study seeks to identify the main factors affecting the health of Quzhou’s registered residents (hukou) and to provide an evidence base for targeted health policies and resource allocation in Quzhou and other similar underdeveloped, aging regions.

## 2 Methods

### 2.1 Data sources

The death data was derived from the registered death cases of registered residents (hukou) in Quzhou, obtained from the vital statistics module of the Zhejiang Province Chronic Disease Monitoring Information Management System. This data was based on the “Medical Certificate of Death (Inference)” completed by medical and health institutions at all administrative levels. Population data was sourced from the full age chart and birth statistics provided by the Public Security Department of Zhejiang Province for Quzhou. Residents in the death and population data refer to individuals registered in the household registration (hukou) system in Quzhou.

Utilizing the Tenth Revision of the International Classification of Diseases (ICD-10) standard for disease classification (15), the top 10 causes of death for different years were ranked based on disease classification (excluding unspecified causes and other diseases). The Chiang abridged life table was constructed using Excel 2019, with 19 age groups: 0-, 1-, 5-, 10-, 15-, 20-, 25-, 30-, 35-, 40-, 45-, 50-, 55-, 60-, 65-, 70-, 75-, 80-, and 85 + years. The population at age 0 was set to the number of live births in the year, while life expectancy for the other age groups was calculated using the average population.

### 2.2 Arriaga decomposition method

The Arriaga decomposition method is used to decompose the differences in life expectancy between different periods, breaking down the difference in life expectancy (i.e., total effect) into three components: direct effect, indirect effect, and interactive effect. With the additivity of the total effect across different age groups, the contributions of different age groups to the increase or decrease in life expectancy are analyzed (2). Calculation formula:


$$\text{Total effect}{:}_{n}TE_{x}{=}_{n}DE_{x}{+}_{n}OE_{x}$$

$$\text{Direct effect}:\mathrm{nD}E_{x}=\frac{l_{x}^{1}(ne_{x}^{2}-ne_{x}^{1})}{l_{0}^{1}}=\frac{l_{x}^{1}}{l_{0}^{1}}\times (\frac{T_{x}^{2}-T_{x+n}^{2}}{l_{x}^{2}}-\frac{T_{x}^{1}-T_{x+n}^{1}}{l_{x}^{1}})$$

$$\text{Direct effect}\;\mathrm{on}\;\text{the open}\;\mathrm{age}\;\text{group}{:}_{n}DE_{x+}=\frac{l_{x}^{1}}{l_{0}^{1}}\times (\frac{T_{x}^{2}}{l_{x}^{2}}-\frac{T_{x}^{1}}{l_{x}^{1}})$$

$$\text{Indirect and interactive effect}{:}_{n}OE_{x}=\frac{T_{x+n}^{2}}{l_{0}^{1}}\times (\frac{l_{x}^{1}}{l_{x}^{2}}-\frac{l_{x+n}^{1}}{l_{x+n}^{2}})$$

*e* represents life expectancy, *l* and *T* refer to the number of survivors at a given age in the life table and the total person-years lived, respectively. 1 and 2 denote population (period) 1 and 2, *x* is the initial age, and *n* is the interval age between groups.

The Arriaga cause decomposition method assumes that the mortality rate at a certain age is equal to the sum of mortality rates from different causes in that age group. It posits that the impact of changes in cause-specific mortality rates within the age interval [*x*, *x* + *n*] on life expectancy is proportional to the total mortality rate of that age group (2). Calculation formula:


$$nk_{\mathrm{xi}}=\frac{{R_{\mathrm{xi}}^{2}}_{n}m_{x}^{2}-{R_{\mathrm{xi}}^{1}}_{n}m_{x}^{1}}{nm_{x}^{2}-nm_{x}^{1}}$$

$$\mathrm{nT}E_{\mathrm{xi}}=\mathrm{nT}E_{x}\times nk_{\mathrm{xi}}$$

*_n_R_xi_* is the ratio of deaths due to a specific cause *i* from age *x* to *x* + *n* in Population (Period) 1 or 2 to total deaths. *_n_m_x_* represents the mortality rate of the population aged [*x*, *x* + *n*].

### 2.3 Quality control

In strict accordance with the “Regulations on the Management of Population Death Information Registration (Trial)” issued by the National Health and Family Planning Commission, all levels of medical institutions in Quzhou report deaths in real-time and undergo three-level quality audits by medical institutions, district/county Centers for Disease Control and Prevention (CDC), and municipal CDC to achieve real-time quality control of death cases. The municipal CDC periodically conducts sample reviews of death cases reported by district/county CDCs and medical institutions to ensure the accuracy of death information reporting. The municipal CDC regularly compares data with multiple departments such as Civil Affairs Bureau, Public Security Bureau, Maternal and Child Health Hospital, and supplements the reporting of any missed death cases.

### 2.4 Statistical analysis

Data management was conducted in R 4.3.2 to establish the database and generate age- and cause-specific death counts, using a standardized cleaning protocol comprising (1) missingness screening with targeted follow-up to complete required fields, (2) outlier and range checks, and (3) internal cross-field logic validation. Life expectancy at birth, $e_{0}^{0}$ , was estimated using a Chiang abridged life table constructed in Excel 2019. Cause-eliminated life expectancy at birth for cause *i*, $e_{0}^{-i}$ , was calculated by removing deaths from cause *i* from the mortality schedule and recomputing the life table. Loss of life expectancy due to cause *i* was defined as $e_{0}^{-i}-e_{0}^{0}$ , and the corresponding loss of life expectancy rate as ( $e_{0}^{-i}-e_{0}^{0}$ )/ $e_{0}^{0}$ × 100% (16). Differences in life expectancy between years were analyzed using the Arriaga decomposition method, with the contribution rate defined as the total effect of each age group divided by the overall change in life expectancy × 100%.

## 3 Results

### 3.1 Age group decomposition of life expectancy growth in Quzhou, 2015–2023

**Figure 1:**
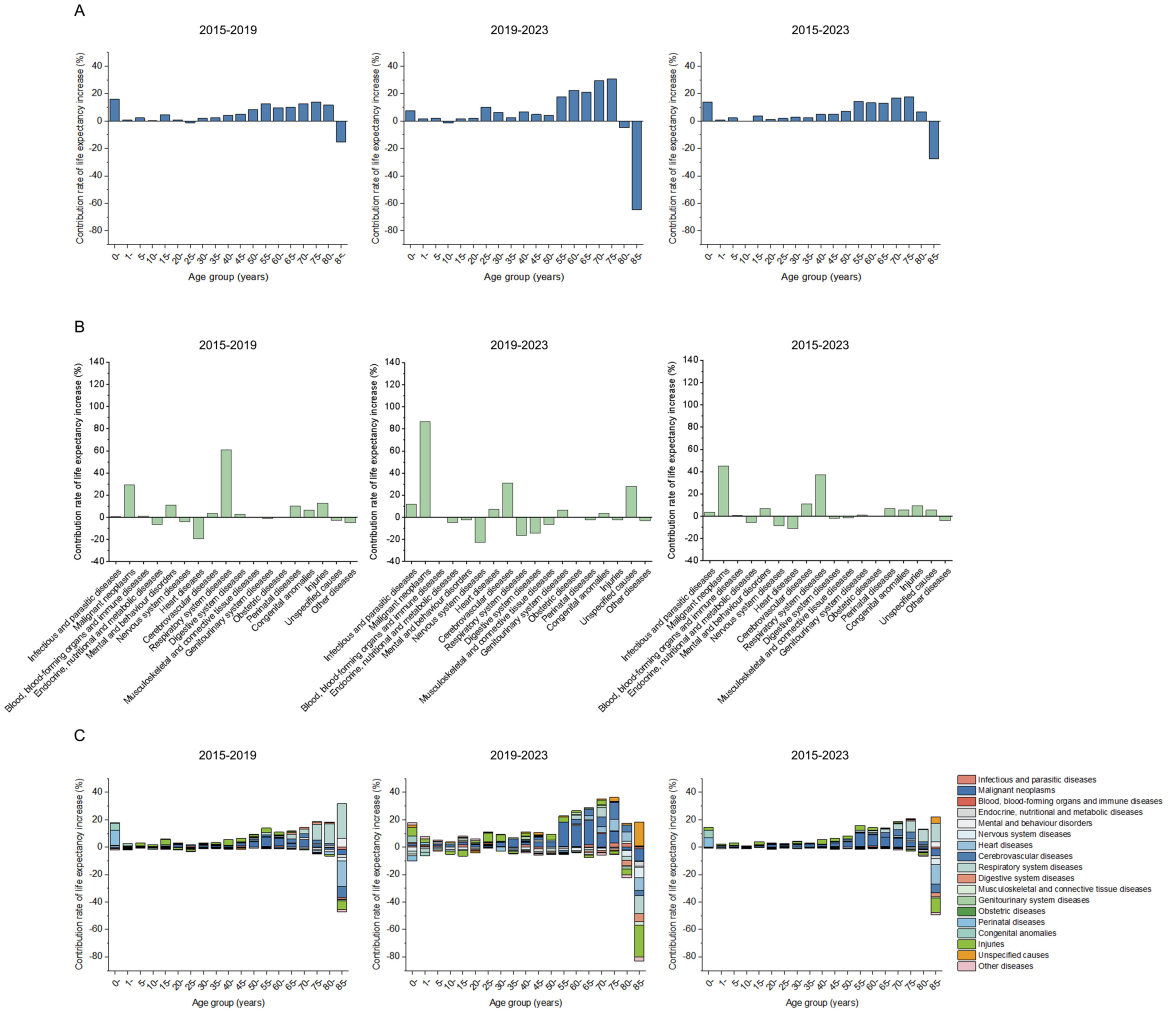
**(A)** Age group decomposition, **(B)** Cause decomposition, and **(C)** Age-specific cause decomposition of life expectancy growth in Quzhou, 2015–2023.

In 2015, 2019, and 2023, the life expectancy in Quzhou was 81.19, 82.31, and 82.74 years, respectively. Over the period from 2015 to 2023, life expectancy increased by 1.55 years, with a growth of 1.12 years from 2015 to 2019, and 0.43 years from 2019 to 2023 (Table 1). Except for the age groups 10- and 85-, all age groups contributed positively to the increase in life expectancy from 2015 to 2023. The top five contributing age groups were 75- (17.77%), 70- (16.78%), 55- (14.07%), 0- (13.72%), and 60- (13.20%). As shown in Figure 1A, from both 2015–2019 and 2019–2023, age groups between 55 and 80 consistently maintained high contribution rates, whereas those between 1 and 20 maintained low contribution rates. Notably, the 0- age group consistently made a positive contribution in both 2015–2019 and 2019–2023; however, its contribution rate in 2019–2023 was 8.42% lower than in 2015–2019. In contrast, the 85 + age group consistently made a negative contribution in both periods, with its contribution rate in 2019–2023 further declining by 49.25% relative to 2015–2019. Based on the sex-specific Arriaga decomposition in Table 2, from 2015 to 2023 the contributions of the 1- and 10- age groups to life expectancy growth were opposite between males and females. In males, the 1- and 10- age groups showed negative contributions (−3.71% and −1.11%, respectively), whereas in females both were positive (6.18 and 0.63%). Similarly, opposite contributions by sex were observed for the 1-, 10-, and 35- age groups in 2015–2019, and for the 1-, 5-, 10-, and 80- age groups in 2019–2023 (Supplementary Tables S1, S2).

**Table 1:** The contribution of changes in mortality rates across different age groups on the increase in life expectancy in Quzhou, 2015–2023.

Age group (years)	2015–2019				2019–2023				2015–2023			
	Direct effect	Indirect and interactive effect	Total effect	Contribution rate (%)	Direct effect	Indirect and interactive effect	Total effect	Contribution rate (%)	Direct effect	Indirect and interactive effect	Total effect	Contribution rate (%)
0-	0.0019	0.1784	0.1803	15.99	0.0003	0.0322	0.0325	7.57	0.0022	0.2115	0.2136	13.72
1-	0.0002	0.0066	0.0067	0.60	0.0002	0.0068	0.0070	1.63	0.0003	0.0134	0.0138	0.88
5-	0.0009	0.0249	0.0258	2.28	0.0003	0.0093	0.0096	2.23	0.0012	0.0343	0.0354	2.28
10-	0.0001	0.0015	0.0016	0.14	−0.0002	−0.0058	−0.0060	−1.40	−0.0002	−0.0043	−0.0044	−0.28
15-	0.0019	0.0474	0.0493	4.37	0.0002	0.0060	0.0063	1.46	0.0021	0.0537	0.0558	3.58
20-	0.0004	0.0104	0.0108	0.96	0.0003	0.0081	0.0085	1.97	0.0008	0.0186	0.0194	1.24
25-	−0.0006	−0.0133	−0.0139	−1.24	0.0019	0.0413	0.0433	10.08	0.0013	0.0278	0.0291	1.87
30-	0.0011	0.0204	0.0215	1.90	0.0013	0.0255	0.0268	6.24	0.0024	0.0459	0.0483	3.10
35-	0.0015	0.0253	0.0268	2.37	0.0005	0.0091	0.0097	2.25	0.0020	0.0346	0.0365	2.35
40-	0.0028	0.0434	0.0462	4.10	0.0018	0.0275	0.0292	6.81	0.0046	0.0711	0.0757	4.86
45-	0.0040	0.0533	0.0573	5.08	0.0014	0.0196	0.0211	4.90	0.0054	0.0732	0.0786	5.05
50-	0.0074	0.0860	0.0934	8.28	0.0014	0.0169	0.0183	4.27	0.0089	0.1034	0.1123	7.21
55-	0.0133	0.1305	0.1438	12.75	0.0069	0.0683	0.0753	17.53	0.0202	0.1990	0.2192	14.07
60-	0.0122	0.0980	0.1102	9.77	0.0106	0.0859	0.0966	22.48	0.0226	0.1828	0.2055	13.20
65-	0.0153	0.0984	0.1137	10.09	0.0123	0.0789	0.0912	21.23	0.0273	0.1754	0.2027	13.02
70-	0.0236	0.1170	0.1406	12.47	0.0215	0.1047	0.1262	29.37	0.0445	0.2168	0.2612	16.78
75-	0.0329	0.1225	0.1554	13.78	0.0292	0.1032	0.1324	30.83	0.0609	0.2157	0.2766	17.77
80-	0.0350	0.0971	0.1321	11.72	−0.0058	−0.0147	−0.0205	−4.78	0.0296	0.0751	0.1047	6.72
85-	−0.1739	0.0000	−0.1739	−15.42	−0.2778	0.0000	−0.2778	−64.67	−0.4270	0.0000	−0.4270	−27.42
Total	−0.0201	1.1477	1.1276	100.00	−0.1934	0.6229	0.4295	100.00	−0.1908	1.7479	1.5571	100.00

**Table 2:** The contribution of changes in mortality rates by sex and age group on the increase in life expectancy in Quzhou, 2015–2023.

Age group (years)	Male				Female				Total			
	Direct effect	Indirect and interactive effect	Total effect	Contribution rate (%)	Direct effect	Indirect and interactive effect	Total effect	Contribution rate (%)	Direct effect	Indirect and interactive effect	Total effect	Contribution rate (%)
0-	0.0026	0.2393	0.2419	15.87	0.0018	0.1791	0.1809	11.88	0.0022	0.2115	0.2136	13.72
1-	−0.0015	−0.0551	−0.0566	−3.71	0.0023	0.0919	0.0942	6.18	0.0003	0.0134	0.0138	0.88
5-	0.0015	0.0415	0.0430	2.82	0.0009	0.0262	0.0270	1.78	0.0012	0.0343	0.0354	2.28
10-	−0.0006	−0.0163	−0.0170	−1.11	0.0003	0.0093	0.0096	0.63	−0.0002	−0.0043	−0.0044	−0.28
15-	0.0024	0.0582	0.0606	3.97	0.0019	0.0486	0.0505	3.31	0.0021	0.0537	0.0558	3.58
20-	0.0008	0.0171	0.0179	1.17	0.0008	0.0184	0.0191	1.26	0.0008	0.0186	0.0194	1.24
25-	0.0018	0.0370	0.0388	2.54	0.0008	0.0184	0.0192	1.26	0.0013	0.0278	0.0291	1.87
30-	0.0033	0.0619	0.0652	4.28	0.0015	0.0304	0.0319	2.09	0.0024	0.0459	0.0483	3.10
35-	0.0036	0.0597	0.0633	4.15	0.0002	0.0038	0.0041	0.27	0.0020	0.0346	0.0365	2.35
40-	0.0063	0.0920	0.0983	6.45	0.0027	0.0443	0.0470	3.08	0.0046	0.0711	0.0757	4.86
45-	0.0055	0.0706	0.0761	4.99	0.0052	0.0753	0.0805	5.29	0.0054	0.0732	0.0786	5.05
50-	0.0095	0.1045	0.1140	7.48	0.0081	0.1010	0.1090	7.16	0.0089	0.1034	0.1123	7.21
55-	0.0282	0.2588	0.2870	18.83	0.0108	0.1152	0.1260	8.27	0.0202	0.1990	0.2192	14.07
60-	0.0271	0.2021	0.2292	15.04	0.0178	0.1565	0.1743	11.44	0.0226	0.1828	0.2055	13.20
65-	0.0206	0.1214	0.1420	9.32	0.0323	0.2265	0.2587	16.98	0.0273	0.1754	0.2027	13.02
70-	0.0407	0.1816	0.2223	14.58	0.0390	0.2088	0.2478	16.27	0.0445	0.2168	0.2612	16.78
75-	0.0659	0.2115	0.2775	18.20	0.0442	0.1724	0.2165	14.22	0.0609	0.2157	0.2766	17.77
80-	0.0168	0.0381	0.0549	3.60	0.0515	0.1444	0.1960	12.87	0.0296	0.0751	0.1047	6.72
85-	−0.4341	0.0000	−0.4341	−28.48	−0.3691	0.0000	−0.3691	−24.23	−0.4270	0.0000	−0.4270	−27.42
Total	−0.1996	1.7238	1.5242	100.00	−0.1472	1.6704	1.5232	100.00	−0.1908	1.7479	1.5571	100.00

### 3.2 Cause decomposition of life expectancy growth in Quzhou, 2015–2023

As shown in Figure 1B, from 2015 to 2019, the main diseases contributing to the increase in life expectancy due to changes in mortality rates were respiratory system diseases (60.81%), malignant neoplasms (29.30%), injuries (12.69%), mental and behavior disorders (10.98%), and perinatal diseases (10.25%). However, from 2019 to 2023, the leading contributors shifted to malignant neoplasms (86.40%), cerebrovascular diseases (30.75%), infectious and parasitic diseases (11.88%), heart diseases (7.36%), and genitourinary system diseases (6.47%). Throughout both four-year periods, malignant neoplasms consistently ranked in the top two contributors and were the highest contributor to the overall increase in life expectancy from 2015 to 2023, at 45.02%. Malignant neoplasms showed the most significant positive growth in contribution rates, increasing by 57.10%, while respiratory diseases exhibited the most notable negative growth, decreasing by 77.07%, shifting from a positive contribution in 2015–2019 to a negative one in 2019–2023. Additionally, infectious and parasitic diseases, cerebrovascular diseases, heart diseases, and genitourinary system diseases also showed substantial growth in contribution rates, increasing by 11.38, 27.19, 26.36, and 7.54%, respectively. Stratified by sex, the diseases contributing to life expectancy gains from 2015 to 2023 differed between males and females. Among males, malignant neoplasms contributed the most (55.23%), whereas among females, respiratory system diseases contributed the most (56.21%), as detailed in Supplementary Figure S1.

### 3.3 Age-specific cause decomposition of life expectancy growth in Quzhou, 2015–2023

According to Figure 1C, the comprehensive contribution rate of the 0- age group to the increase in life expectancy was 13.72% from 2015 to 2023. The combined contribution rate of perinatal diseases (6.78%), congenital anomalies (5.27%), and injuries (2.03%) was 1.03 times the comprehensive contribution rate of the 0- age group. Consistent with previous findings, the contribution rates of the 55 to 80 age groups to the increase in life expectancy from 2015 to 2023 remained high. The five age groups involved were 55- (14.07%), 60- (13.20%), 65- (13.02%), 70- (16.78%), and 75- (17.77%). Among these age groups, the contribution of malignant neoplasms to the comprehensive contribution rate of each age group was 68.75, 62.58, 50.67, 37.67, and 19.19%, showing a decreasing trend with increasing age. The 85- age group negatively impacted the increase in life expectancy from 2015 to 2023, with a comprehensive contribution rate of −27.42%. The three diseases with the lowest contribution rates in this age group were heart disease (−14.71%), injuries (−10.39%), and cerebrovascular diseases (−6.33%).

### 3.4 Changes of death spectrum by diseases in Quzhou, 2015–2023

From 2015 to 2023, there were some changes in the top 10 causes of death in Quzhou. In 2019, genitourinary system diseases replaced mental and behavior disorders from 2015 in the top 10, while in 2023, mental and behavior disorders again replaced genitourinary system diseases. Over these three years, malignant neoplasms consistently ranked as the leading cause of death. Respiratory diseases dropped from the second position in 2015 to the third in 2019, then rose back to the second position in 2023. The ranking of cerebrovascular diseases showed the opposite trend, first rising and then falling. In both 2019 and 2023, the rankings for heart diseases and injuries remained unchanged, at fourth and fifth, respectively (Table 3).

**Table 3:** Changes of death spectrum by diseases in Quzhou, 2015–2023.

Order of ranking	2015			2019			2023		
	Diseases	Mortality rate (per 100,000)	Composition ratio (%)	Diseases	Mortality rate (per 100,000)	Composition ratio (%)	Diseases	Mortality rate (per 100,000)	Composition ratio (%)
1	Malignant neoplasms	179.96	27.14	Malignant neoplasms	183.17	26.91	Malignant neoplasms	189.82	24.81
2	Respiratory system diseases	137.44	20.73	Cerebrovascular diseases	116.61	17.13	Respiratory system diseases	126.52	16.54
3	Cerebrovascular diseases	107.00	16.14	Respiratory system diseases	106.03	15.58	Cerebrovascular diseases	125.57	16.41
4	Injuries	74.88	11.29	Heart diseases	86.08	12.65	Heart diseases	95.84	12.53
5	Heart diseases	62.93	9.49	Injuries	77.72	11.42	Injuries	93.34	12.20
6	Digestive system diseases	15.46	2.33	Endocrine, nutritional and metabolic diseases	21.59	3.17	Endocrine, nutritional and metabolic diseases	26.36	3.45
7	Endocrine, nutritional and metabolic diseases	14.98	2.26	Infectious and parasitic diseases	15.03	2.21	Digestive system diseases	22.39	2.93
8	Mental and behavior disorders	14.18	2.14	Digestive system diseases	14.79	2.17	Nervous system diseases	20.93	2.74
9	Infectious and parasitic diseases	13.58	2.05	Nervous system diseases	12.33	1.81	Infectious and parasitic diseases	14.67	1.92
10	Nervous system diseases	8.55	1.29	Genitourinary system diseases	8.59	1.26	Mental and behavior disorders	9.83	1.28

### 3.5 Changes of cause-eliminated life expectancy by diseases in Quzhou, 2015–2023

As shown in Table 4, the loss of life expectancy rate due to malignant neoplasms consistently ranked first at 4.22, 3.89, and 3.32% in 2015, 2019, and 2023, respectively. The loss of life expectancy due to respiratory diseases decreased from 3.22 years in 2015 to 1.92 years in 2023, with the loss of life expectancy rate dropping from 3.97 to 2.32%. From 2015 to 2023, besides malignant neoplasms and respiratory diseases, the loss of life expectancy rate for injuries also consistently declined. Diseases with consistently increasing loss of life expectancy rate included endocrine, nutritional and metabolic diseases, as well as nervous system diseases. Additionally, cerebrovascular and heart diseases showed a rise-then-fall trend, while digestive system diseases showed a fall-then-rise trend in the loss of life expectancy rate. As shown in Supplementary Figures S3–S5, there are marked differences in the ranking of life expectancy loss rates by sex. In 2015, the leading cause of life expectancy loss among males was malignant neoplasms, consistent with the total population, whereas among females it was respiratory system diseases. By 2019 and 2023, the top five causes for males remained unchanged, while the female ranking shifted, with malignant neoplasms, cerebrovascular diseases, and heart diseases surpassing respiratory system diseases in life expectancy loss rate.

**Table 4:** Changes of cause-eliminated life expectancy by diseases in Quzhou, 2015–2023.

Order of ranking	2015				2019				2023			
	Diseases	$e_{0}^{-i}$	$e_{0}^{-i}-e_{0}^{0}$	$(e_{0}^{-i}-e_{0}^{0})/e_{0}^{-i}$ (%)	Diseases	$e_{0}^{-i}$	$e_{0}^{-i}-e_{0}^{0}$	$(e_{0}^{-i}-e_{0}^{0})/e_{0}^{-i}$ (%)	Diseases	$e_{0}^{-i}$	$e_{0}^{-i}-e_{0}^{0}$	$(e_{0}^{-i}-e_{0}^{0})/e_{0}^{-i}$ (%)
1	Malignant neoplasms	84.62	3.43	4.22	Malignant neoplasms	85.51	3.20	3.89	Malignant neoplasms	85.49	2.75	3.32
2	Respiratory system diseases	84.41	3.22	3.97	Cerebrovascular diseases	84.38	2.07	2.51	Respiratory system diseases	84.66	1.92	2.32
3	Cerebrovascular diseases	83.22	2.03	2.50	Respiratory system diseases	84.29	1.98	2.41	Cerebrovascular diseases	84.54	1.80	2.18
4	Injuries	82.81	1.62	2.00	Injuries	83.85	1.54	1.87	Injuries	84.27	1.53	1.85
5	Heart diseases	82.34	1.15	1.42	Heart diseases	83.83	1.52	1.85	Heart diseases	84.12	1.38	1.67
6	Digestive system diseases	81.45	0.26	0.32	Endocrine, nutritional and metabolic diseases	82.65	0.34	0.41	Endocrine, nutritional and metabolic diseases	83.09	0.35	0.42
7	Endocrine, nutritional and metabolic diseases	81.44	0.25	0.31	Infectious and parasitic diseases	82.56	0.25	0.30	Nervous system diseases	83.05	0.31	0.37
8	Mental and behavior disorders	81.44	0.25	0.31	Digestive system diseases	82.55	0.24	0.29	Digestive system diseases	83.04	0.30	0.36
9	Infectious and parasitic diseases	81.43	0.24	0.30	Nervous system diseases	82.53	0.22	0.27	Infectious and parasitic diseases	82.93	0.19	0.23
10	Nervous system diseases	81.35	0.16	0.20	Genitourinary system diseases	82.46	0.15	0.18	Mental and behavior disorders	82.87	0.13	0.16

## 4 Discussion

The analysis using the Arriaga decomposition method provided insightful perspectives on the changes in life expectancy in Quzhou from 2015 to 2023. By breaking down the differences in life expectancy into direct, indirect, and interaction effects, we gained a nuanced understanding of the contributions made by different age groups.

From 2015 to 2023, life expectancy in Quzhou increased steadily from 81.19 to 82.74 years, with a total gain of 1.55 years. The increase was more pronounced during 2015–2019 (1.12 years) than in 2019–2023 (0.43 years), indicating a deceleration in recent gains. This slowdown may reflect a natural plateau effect as overall health status improves, as well as external influences such as population aging and the lingering impact of COVID-19 on healthcare service delivery (17, 18). Individuals aged 55–80 were the main contributors to the increase in life expectancy, accounting for over 60% of the total gain. Since 2015, Zhejiang Province has implemented standardized management of hypertension and diabetes at the grass-roots level, and more recently expanded to include dyslipidemia and chronic obstructive pulmonary disease (COPD). Initiatives such as family-doctor contracting, enhancement of basic public health services, and older adults health services may have driven the larger contribution of the 55–80 age group (19). Interestingly, the 0- age group, typically associated with infant mortality, also showed a positive contribution to life expectancy, although the magnitude of this contribution declined from 2019 to 2023. A research on life expectancy decomposition in Zhejiang Province observed similar results, with the contribution rate of the infant group declining continuously from 2011 to 2020 (12). The negative contribution of the 85- age group to life expectancy was prominent, indicating that the oldest-old population remains high vulnerability in cardiovascular, respiratory, neurodegenerative, and frailty syndromes. Based on domestic and foreign research, strategies for active aging and delayed disability (such as comprehensive older adults assessment, fall intervention, vaccination, home and long-term care service supply, and improvement of end-of-life care quality) are particularly crucial in offsetting the negative pull of the oldest-old population (20–24).

In 2015–2019, respiratory diseases, malignant neoplasms, and injuries were the main contributors to increased life expectancy; In 2019–2023, malignant neoplasms became the leading contributor, followed by cerebrovascular and infectious diseases. A comparison of the two periods revealed a notable rise in the contribution of malignant neoplasms, while respiratory diseases shifted from a positive to a negative contributor. This pattern is consistent with pandemic-era changes in the mortality structure observed both domestically and internationally: multiple analyses have documented substantial excess mortality concentrated at older ages during COVID-19 waves. For example, according to the WHO, the total number of deaths directly or indirectly attributable to the COVID-19 pandemic (excess deaths) was approximately 14.9 million in 2020 and through 2021 (25). A U.S. cohort study indicated that although annual excess deaths peaked in 2021, more than 1.5 million excess deaths still occurred during 2022–2023. In 2023, the excess mortality rate remained substantially higher than pre-pandemic levels (26). In addition, many studies show that COVID-19 itself directly led to medium- and long-term respiratory health problems and may have increased respiratory vulnerability in some populations (27). Consequently, the incidence and mortality of respiratory diseases were higher after the pandemic than before it; in our study, we also found that respiratory mortality in 2023 exceeded that of 2019. In contrast, the gradual recovery of chronic disease management and the promotion of early cancer screening amplified improvements in neoplasm-related mortality (28, 29). Throughout the study period, malignant neoplasms contributed the most to the overall increase in life expectancy (45.02%), with high impact in the 55–75 age group. This reflects the effectiveness of tumor screening and treatment in middle-aged and older populations. Screening programs for colorectal and breast cancer in Quzhou, along with Zhejiang Province’s early diagnosis and treatment initiative and strengthened tumor care under the hierarchical medical system, may have played a pivotal role.

Sex-stratified Arriaga decompositions by age group and cause show that males and females contributed differently to life expectancy gains, with some younger age groups (e.g., 1- and 10-) exhibiting opposite contributions. This pattern likely reflects small death counts and resultant volatility within narrow child age bands. Moreover, the cause-specific decomposition revealed a notable contrast for 2015–2023: malignant neoplasms contributed most to life expectancy gains in males, whereas respiratory system diseases did so in females, indicating distinct sex-specific contribution profiles.

The 0-year age group contributed 13.72% to the overall gain in life expectancy, with perinatal conditions, congenital anomalies, and injuries making slightly higher contributions than the age group’s overall share. This highlights the importance of continuously improving maternal and child health services, preventing congenital defects, and reducing infant injuries. The 55–80 age range is identified as a “golden window period” for modifiable health outcomes (30, 31). Cause-specific decomposition shows that malignant neoplasms are the largest contributors within this group, with impact gradually declining with age, indicating that early detection and standardized treatment are more meaningful at younger age group. The negative contributions of the 85- age group primarily stem from heart disease, injury, and cerebrovascular disease, suggesting that fall and aspiration prevention, blood pressure and lipid control, rehabilitation, and long-term care should be regarded as essential health strategies for the older adults (32–36).

Changes of death spectrum by diseases in Quzhou from 2015 to 2023 reflected shifts in population structure, socio-economic conditions, healthcare quality, and health awareness. Firstly, malignant neoplasms had consistently ranked first in the cause of death, indicating that cancer remained the leading cause of disease burden in Quzhou. This trend may have been closely related to aging, lifestyle changes (such as smoking and dietary habits), and environmental factors (4, 37, 38). The fluctuation in the ranking of respiratory diseases highlighted the variable nature of these diseases. From 2015 to 2019, the life expectancy lost due to respiratory diseases steadily decreased, reflecting advancements in the prevention and treatment of such conditions. However, the ranking of respiratory diseases rose from the third place in 2019 to the second place in 2023, and the mortality rate rose from 106.03/100000 to 126.52/100000, which may be affected by seasonal diseases such as COVID-19, influenza or pneumonia (18, 28, 29). The changes in the rankings of cerebrovascular diseases and heart diseases were also noteworthy. Cerebrovascular disease showed a rise and then a decline in ranking, while heart disease remained relatively stable. This might indicate improvements in managing and treating cardiovascular diseases, but cerebrovascular diseases remained an area requiring further attention, especially in terms of prevention and rehabilitation. Additionally, the rate of life expectancy loss from endocrine, nutritional, and metabolic diseases and nervous system diseases has been on a steady rise, indicating a growing burden of chronic conditions—including diabetes, obesity-related complications, and chronic neurodegenerative diseases such as dementia and Parkinson’s disease. This was consistent with the global epidemic trend of non-communicable diseases (NCDs) (21, 23, 39).

The limitations of this study include: (1) The death and population data used for life expectancy calculations were based on registered residents (hukou) rather than usual residents, excluding the lifestyle, health conditions, and healthcare resource utilization of mobile populations, which may differ from registered residents (hukou) and introduce bias to the life expectancy calculations (12); (2) Delays in household registration and deregistration in the public security system may lead to errors in population data, thereby affecting life expectancy calculations; (3) Life expectancy, as a comprehensive indicator, the data in this study cannot delve into the specific influencing factors behind the changes in life expectancy, such as economic development level, behavioral lifestyles, and environmental factors (3).

To continue providing health policy guidance for underdeveloped, rapidly aging areas like Quzhou, we will pursue longitudinal, individual-level studies by linking electronic health records, chronic disease registries, and mortality registers. This will enable assessment of how specific exposures (e.g., hypertension diagnosis, cancer screening, COVID-19 infection) affect cause-specific mortality. We will also apply Joinpoint regression to identify trend inflection points (e.g., around 2020), followed by segmented or multivariable models to compare determinants across periods and inform targeted policies and resource allocation.

## 5 Conclusion

The analysis of life expectancy and mortality trends in Quzhou from 2015 to 2023 revealed a complex interplay of factors that influenced health outcomes in the region. Overall, these findings suggested the need for comprehensive healthcare policies that addressed the specific needs of different age groups and disease burdens. Enhancing access to healthcare services, promoting healthy aging initiatives, and tailoring interventions for chronic conditions were crucial for sustaining and improving population health in Quzhou. By understanding these patterns and implementing evidence-based strategies, public health efforts could continue to drive positive health outcomes in the region.

## Data Availability

The raw data supporting the conclusions of this article will be made available by the authors, without undue reservation.
