# Supplementary figures and images for: Unraveling life expectancy and death spectrum changes of registered residents (hukou) in Quzhou, China, 2015–2023: a study using Arriaga decomposition method
Source: Front Public Health. 2025 Nov 28;13:1687798. doi: 10.3389/fpubh.2025.1687798 (PMC12698370; doi:10.3389/fpubh.2025.1687798)

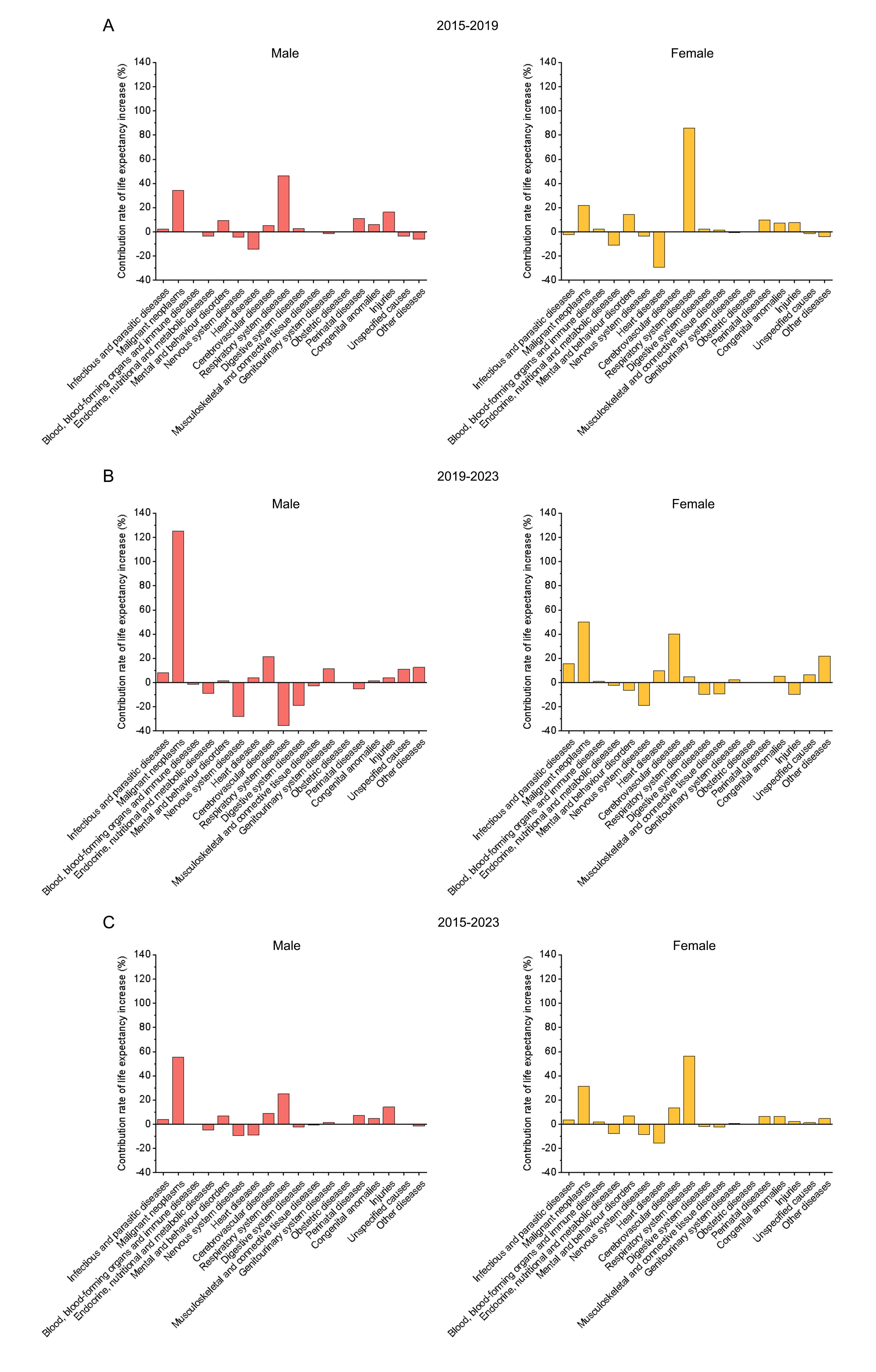

Supplement: Supplementary file 1 [file Image_1.JPEG]
