# Supplementary material for: Unraveling life expectancy and death spectrum changes of registered residents (hukou) in Quzhou, China, 2015–2023: a study using Arriaga decomposition method
Source: Front Public Health. 2025 Nov 28;13:1687798. doi: 10.3389/fpubh.2025.1687798 (PMC12698370; doi:10.3389/fpubh.2025.1687798)
Supplement: Supplementary file 2 [file Table_1.DOCX]

**Table S1.** The contribution of changes in mortality rates by sex and age group on the increase in life expectancy in Quzhou, 2015-2019.

| Age group （years） | Male | | | |  | Female | | | |  | Total | | | |
| --- | --- | --- | --- | --- | --- | --- | --- | --- | --- | --- | --- | --- | --- | --- |
|  | Direct effect | Indirect and interactive effect | Total effect | Contribution rate (%) |  | Direct effect | Indirect and interactive effect | Total effect | Contribution rate (%) |  | Direct effect | Indirect and interactive effect | Total effect | Contribution rate (%) |
| 0- | 0.0023 | 0.2116 | 0.2139 | 18.29 |  | 0.0014 | 0.1403 | 0.1418 | 14.14 |  | 0.0019 | 0.1784 | 0.1803 | 15.99 |
| 1- | -0.0012 | -0.0444 | -0.0455 | -3.90 |  | 0.0016 | 0.0646 | 0.0662 | 6.60 |  | 0.0002 | 0.0066 | 0.0067 | 0.60 |
| 5- | 0.0005 | 0.0143 | 0.0148 | 1.26 |  | 0.0012 | 0.0369 | 0.0381 | 3.80 |  | 0.0009 | 0.0249 | 0.0258 | 2.28 |
| 10- | 0.0005 | 0.0135 | 0.0140 | 1.20 |  | -0.0004 | -0.0121 | -0.0126 | -1.25 |  | 0.0001 | 0.0015 | 0.0016 | 0.14 |
| 15- | 0.0019 | 0.0469 | 0.0488 | 4.18 |  | 0.0018 | 0.0479 | 0.0497 | 4.96 |  | 0.0019 | 0.0474 | 0.0493 | 4.37 |
| 20- | 0.0006 | 0.0143 | 0.0150 | 1.28 |  | 0.0002 | 0.0041 | 0.0043 | 0.43 |  | 0.0004 | 0.0104 | 0.0108 | 0.96 |
| 25- | -0.0010 | -0.0214 | -0.0224 | -1.92 |  | -0.0001 | -0.0028 | -0.0029 | -0.29 |  | -0.0006 | -0.0133 | -0.0139 | -1.24 |
| 30- | 0.0012 | 0.0218 | 0.0230 | 1.97 |  | 0.0009 | 0.0182 | 0.0191 | 1.91 |  | 0.0011 | 0.0204 | 0.0215 | 1.90 |
| 35- | 0.0039 | 0.0646 | 0.0685 | 5.86 |  | -0.0011 | -0.0203 | -0.0214 | -2.14 |  | 0.0015 | 0.0253 | 0.0268 | 2.37 |
| 40- | 0.0037 | 0.0541 | 0.0578 | 4.94 |  | 0.0018 | 0.0297 | 0.0315 | 3.14 |  | 0.0028 | 0.0434 | 0.0462 | 4.10 |
| 45- | 0.0051 | 0.0644 | 0.0694 | 5.94 |  | 0.0028 | 0.0398 | 0.0426 | 4.25 |  | 0.0040 | 0.0533 | 0.0573 | 5.08 |
| 50- | 0.0087 | 0.0950 | 0.1037 | 8.87 |  | 0.0060 | 0.0743 | 0.0803 | 8.01 |  | 0.0074 | 0.0860 | 0.0934 | 8.28 |
| 55- | 0.0155 | 0.1419 | 0.1574 | 13.46 |  | 0.0104 | 0.1094 | 0.1197 | 11.94 |  | 0.0133 | 0.1305 | 0.1438 | 12.75 |
| 60- | 0.0139 | 0.1041 | 0.1180 | 10.09 |  | 0.0106 | 0.0925 | 0.1031 | 10.28 |  | 0.0122 | 0.0980 | 0.1102 | 9.77 |
| 65- | 0.0082 | 0.0490 | 0.0572 | 4.90 |  | 0.0207 | 0.1444 | 0.1651 | 16.47 |  | 0.0153 | 0.0984 | 0.1137 | 10.09 |
| 70- | 0.0198 | 0.0911 | 0.1109 | 9.49 |  | 0.0223 | 0.1191 | 0.1413 | 14.10 |  | 0.0236 | 0.1170 | 0.1406 | 12.47 |
| 75- | 0.0464 | 0.1602 | 0.2066 | 17.67 |  | 0.0115 | 0.0459 | 0.0575 | 5.73 |  | 0.0329 | 0.1225 | 0.1554 | 13.78 |
| 80- | 0.0304 | 0.0786 | 0.1090 | 9.32 |  | 0.0444 | 0.1301 | 0.1745 | 17.41 |  | 0.0350 | 0.0971 | 0.1321 | 11.72 |
| 85- | -0.1508 | 0.0000 | -0.1508 | -12.90 |  | -0.1955 | 0.0000 | -0.1955 | -19.50 |  | -0.1739 | 0.0000 | -0.1739 | -15.42 |
| Total | 0.0096 | 1.1596 | 1.1692 | 100.00 |  | -0.0595 | 1.0620 | 1.0025 | 100.00 |  | -0.0201 | 1.1477 | 1.1276 | 100.00 |
