# Supplementary material for: Unraveling life expectancy and death spectrum changes of registered residents (hukou) in Quzhou, China, 2015–2023: a study using Arriaga decomposition method
Source: Front Public Health. 2025 Nov 28;13:1687798. doi: 10.3389/fpubh.2025.1687798 (PMC12698370; doi:10.3389/fpubh.2025.1687798)
Supplement: Supplementary file 3 [file Table_2.DOCX]

**Table S2.** The contribution of changes in mortality rates by sex and age group on the increase in life expectancy in Quzhou, 2019-2023.

| Age group （years） | Male | | | |  | Female | | | |  | Total | | | |
| --- | --- | --- | --- | --- | --- | --- | --- | --- | --- | --- | --- | --- | --- | --- |
|  | Direct effect | Indirect and interactive effect | Total effect | Contribution rate (%) |  | Direct effect | Indirect and interactive effect | Total effect | Contribution rate (%) |  | Direct effect | Indirect and interactive effect | Total effect | Contribution rate (%) |
| 0- | 0.0003 | 0.0268 | 0.0271 | 7.64 |  | 0.0004 | 0.0380 | 0.0383 | 7.36 |  | 0.0003 | 0.0322 | 0.0325 | 7.57 |
| 1- | -0.0003 | -0.0106 | -0.0109 | -3.06 |  | 0.0007 | 0.0270 | 0.0276 | 5.31 |  | 0.0002 | 0.0068 | 0.0070 | 1.63 |
| 5- | 0.0010 | 0.0273 | 0.0282 | 7.95 |  | -0.0004 | -0.0110 | -0.0114 | -2.18 |  | 0.0003 | 0.0093 | 0.0096 | 2.23 |
| 10- | -0.0011 | -0.0300 | -0.0311 | -8.76 |  | 0.0008 | 0.0216 | 0.0223 | 4.29 |  | -0.0002 | -0.0058 | -0.0060 | -1.40 |
| 15- | 0.0005 | 0.0111 | 0.0115 | 3.25 |  | 0.0000 | 0.0004 | 0.0004 | 0.08 |  | 0.0002 | 0.0060 | 0.0063 | 1.46 |
| 20- | 0.0001 | 0.0027 | 0.0028 | 0.80 |  | 0.0006 | 0.0143 | 0.0148 | 2.85 |  | 0.0003 | 0.0081 | 0.0085 | 1.97 |
| 25- | 0.0029 | 0.0587 | 0.0615 | 17.33 |  | 0.0010 | 0.0213 | 0.0222 | 4.27 |  | 0.0019 | 0.0413 | 0.0433 | 10.08 |
| 30- | 0.0022 | 0.0400 | 0.0422 | 11.89 |  | 0.0006 | 0.0121 | 0.0127 | 2.44 |  | 0.0013 | 0.0255 | 0.0268 | 6.24 |
| 35- | -0.0003 | -0.0053 | -0.0056 | -1.57 |  | 0.0013 | 0.0244 | 0.0257 | 4.94 |  | 0.0005 | 0.0091 | 0.0097 | 2.25 |
| 40- | 0.0026 | 0.0379 | 0.0405 | 11.39 |  | 0.0009 | 0.0144 | 0.0153 | 2.93 |  | 0.0018 | 0.0275 | 0.0292 | 6.81 |
| 45- | 0.0005 | 0.0058 | 0.0063 | 1.77 |  | 0.0024 | 0.0353 | 0.0377 | 7.25 |  | 0.0014 | 0.0196 | 0.0211 | 4.90 |
| 50- | 0.0008 | 0.0089 | 0.0097 | 2.74 |  | 0.0021 | 0.0261 | 0.0281 | 5.41 |  | 0.0014 | 0.0169 | 0.0183 | 4.27 |
| 55- | 0.0129 | 0.1181 | 0.1310 | 36.90 |  | 0.0004 | 0.0047 | 0.0051 | 0.98 |  | 0.0069 | 0.0683 | 0.0753 | 17.53 |
| 60- | 0.0135 | 0.1003 | 0.1138 | 32.06 |  | 0.0073 | 0.0639 | 0.0711 | 13.66 |  | 0.0106 | 0.0859 | 0.0966 | 22.48 |
| 65- | 0.0127 | 0.0749 | 0.0876 | 24.68 |  | 0.0117 | 0.0824 | 0.0942 | 18.08 |  | 0.0123 | 0.0789 | 0.0912 | 21.23 |
| 70- | 0.0216 | 0.0962 | 0.1178 | 33.19 |  | 0.0172 | 0.0920 | 0.1092 | 20.96 |  | 0.0215 | 0.1047 | 0.1262 | 29.37 |
| 75- | 0.0203 | 0.0653 | 0.0856 | 24.11 |  | 0.0339 | 0.1321 | 0.1660 | 31.88 |  | 0.0292 | 0.1032 | 0.1324 | 30.83 |
| 80- | -0.0147 | -0.0334 | -0.0481 | -13.54 |  | 0.0075 | 0.0210 | 0.0285 | 5.48 |  | -0.0058 | -0.0147 | -0.0205 | -4.78 |
| 85- | -0.3151 | 0.0000 | -0.3151 | -88.77 |  | -0.1874 | 0.0000 | -0.1874 | -35.99 |  | -0.2778 | 0.0000 | -0.2778 | -64.67 |
| Total | -0.2398 | 0.5948 | 0.3550 | 100.00 |  | -0.0991 | 0.6198 | 0.5208 | 100.00 |  | -0.1934 | 0.6229 | 0.4295 | 100.00 |
