# Supplementary material for: Unraveling life expectancy and death spectrum changes of registered residents (hukou) in Quzhou, China, 2015–2023: a study using Arriaga decomposition method
Source: Front Public Health. 2025 Nov 28;13:1687798. doi: 10.3389/fpubh.2025.1687798 (PMC12698370; doi:10.3389/fpubh.2025.1687798)
Supplement: Supplementary file 4 [file Table_3.DOCX]

**Table S3.** Changes of cause-eliminated life expectancy by diseases among different sexes in Quzhou, 2015.

| Order of ranking | Male | | | |  | Female | | | |  | Total | | | |
| --- | --- | --- | --- | --- | --- | --- | --- | --- | --- | --- | --- | --- | --- | --- |
|  | Diseases | e-i 0 | e-i 0- e0 0 | (e-i 0- e0 0)/e-i 0(%) |  | Diseases | e-i 0 | e-i 0- e0 0 | (e-i 0- e0 0)/e-i 0(%) |  | Diseases | e-i 0 | e-i 0- e0 0 | (e-i 0- e0 0)/e-i 0(%) |
| 1 | Malignant neoplasms | 83.17 | 4.11 | 5.20 |  | Respiratory system diseases | 87.03 | 3.28 | 3.92 |  | Malignant neoplasms | 84.62 | 3.43 | 4.22 |
| 2 | Respiratory system diseases | 82.18 | 3.12 | 3.95 |  | Malignant neoplasms | 86.26 | 2.51 | 3.00 |  | Respiratory system diseases | 84.41 | 3.22 | 3.97 |
| 3 | Cerebrovascular diseases | 80.93 | 1.87 | 2.37 |  | Cerebrovascular diseases | 85.93 | 2.18 | 2.60 |  | Cerebrovascular diseases | 83.22 | 2.03 | 2.50 |
| 4 | Injuries | 80.83 | 1.77 | 2.24 |  | Injuries | 85.20 | 1.45 | 1.73 |  | Injuries | 82.81 | 1.62 | 2.00 |
| 5 | Heart diseases | 80.07 | 1.01 | 1.28 |  | Heart diseases | 85.08 | 1.33 | 1.59 |  | Heart diseases | 82.34 | 1.15 | 1.42 |
| 6 | Digestive system diseases | 79.33 | 0.27 | 0.34 |  | Endocrine, nutritional and metabolic diseases | 84.07 | 0.32 | 0.38 |  | Digestive system diseases | 81.45 | 0.26 | 0.32 |
| 7 | Infectious and parasitic diseases | 79.33 | 0.27 | 0.34 |  | Mental and behaviour disorders | 84.05 | 0.30 | 0.36 |  | Endocrine, nutritional and metabolic diseases | 81.44 | 0.25 | 0.31 |
| 8 | Mental and behaviour disorders | 79.27 | 0.21 | 0.27 |  | Digestive system diseases | 83.99 | 0.24 | 0.29 |  | Mental and behaviour disorders | 81.44 | 0.25 | 0.31 |
| 9 | Endocrine, nutritional and metabolic diseases | 79.26 | 0.20 | 0.25 |  | Nervous system diseases | 83.96 | 0.21 | 0.25 |  | Infectious and parasitic diseases | 81.43 | 0.24 | 0.30 |
| 10 | Genitourinary system diseases | 79.19 | 0.13 | 0.16 |  | Infectious and parasitic diseases | 83.94 | 0.19 | 0.23 |  | Nervous system diseases | 81.35 | 0.16 | 0.20 |
