# Supplementary material for: Unraveling life expectancy and death spectrum changes of registered residents (hukou) in Quzhou, China, 2015–2023: a study using Arriaga decomposition method
Source: Front Public Health. 2025 Nov 28;13:1687798. doi: 10.3389/fpubh.2025.1687798 (PMC12698370; doi:10.3389/fpubh.2025.1687798)
Supplement: Supplementary file 5 [file Table_4.DOCX]

**Table S4.** Changes of cause-eliminated life expectancy by diseases among different sexes in Quzhou, 2019.

| Order of ranking | Male | | | |  | Female | | | |  | Total | | | |
| --- | --- | --- | --- | --- | --- | --- | --- | --- | --- | --- | --- | --- | --- | --- |
|  | Diseases | e-i 0 | e-i 0- e0 0 | (e-i 0- e0 0)/e-i 0(%) |  | Diseases | e-i 0 | e-i 0- e0 0 | (e-i 0- e0 0)/e-i 0(%) |  | Diseases | e-i 0 | e-i 0- e0 0 | (e-i 0- e0 0)/e-i 0(%) |
| 1 | Malignant neoplasms | 84.06 | 3.83 | 4.77 |  | Malignant neoplasms | 87.10 | 2.35 | 2.77 |  | Malignant neoplasms | 85.51 | 3.20 | 3.89 |
| 2 | Respiratory system diseases | 82.35 | 2.12 | 2.64 |  | Cerebrovascular diseases | 87.02 | 2.27 | 2.68 |  | Cerebrovascular diseases | 84.38 | 2.07 | 2.51 |
| 3 | Cerebrovascular diseases | 82.12 | 1.89 | 2.36 |  | Heart diseases | 86.57 | 1.82 | 2.15 |  | Respiratory system diseases | 84.29 | 1.98 | 2.41 |
| 4 | Injuries | 81.87 | 1.64 | 2.04 |  | Respiratory system diseases | 86.49 | 1.74 | 2.05 |  | Injuries | 83.85 | 1.54 | 1.87 |
| 5 | Heart diseases | 81.51 | 1.28 | 1.60 |  | Injuries | 86.17 | 1.42 | 1.68 |  | Heart diseases | 83.83 | 1.52 | 1.85 |
| 6 | Endocrine, nutritional and metabolic diseases | 80.48 | 0.25 | 0.31 |  | Endocrine, nutritional and metabolic diseases | 85.20 | 0.45 | 0.53 |  | Endocrine, nutritional and metabolic diseases | 82.65 | 0.34 | 0.41 |
| 7 | Infectious and parasitic diseases | 80.48 | 0.25 | 0.31 |  | Nervous system diseases | 85.00 | 0.25 | 0.30 |  | Infectious and parasitic diseases | 82.56 | 0.25 | 0.30 |
| 8 | Digestive system diseases | 80.48 | 0.25 | 0.31 |  | Infectious and parasitic diseases | 84.98 | 0.23 | 0.27 |  | Digestive system diseases | 82.55 | 0.24 | 0.29 |
| 9 | Nervous system diseases | 80.42 | 0.19 | 0.24 |  | Digestive system diseases | 84.97 | 0.22 | 0.26 |  | Nervous system diseases | 82.53 | 0.22 | 0.27 |
| 10 | Genitourinary system diseases | 80.39 | 0.16 | 0.20 |  | Mental and behaviour disorders | 84.90 | 0.15 | 0.18 |  | Genitourinary system diseases | 82.46 | 0.15 | 0.18 |
