# Supplementary material for: Unraveling life expectancy and death spectrum changes of registered residents (hukou) in Quzhou, China, 2015–2023: a study using Arriaga decomposition method
Source: Front Public Health. 2025 Nov 28;13:1687798. doi: 10.3389/fpubh.2025.1687798 (PMC12698370; doi:10.3389/fpubh.2025.1687798)
Supplement: Supplementary file 6 [file Table_5.DOCX]

**Table S5.** Changes of cause-eliminated life expectancy by diseases among different sexes in Quzhou, 2023.

| Order of ranking | Male | | | |  | Female | | | |  | Total | | | |
| --- | --- | --- | --- | --- | --- | --- | --- | --- | --- | --- | --- | --- | --- | --- |
|  | Diseases | e-i 0 | e-i 0- e0 0 | (e-i 0- e0 0)/e-i 0(%) |  | Diseases | e-i 0 | e-i 0- e0 0 | (e-i 0- e0 0)/e-i 0(%) |  | Diseases | e-i 0 | e-i 0- e0 0 | (e-i 0- e0 0)/e-i 0(%) |
| 1 | Malignant neoplasms | 83.80 | 3.22 | 4.00 |  | Malignant neoplasms | 87.35 | 2.08 | 2.44 |  | Malignant neoplasms | 85.49 | 2.75 | 3.32 |
| 2 | Respiratory system diseases | 82.63 | 2.05 | 2.54 |  | Cerebrovascular diseases | 87.22 | 1.95 | 2.29 |  | Respiratory system diseases | 84.66 | 1.92 | 2.32 |
| 3 | Cerebrovascular diseases | 82.23 | 1.65 | 2.05 |  | Heart diseases | 86.99 | 1.72 | 2.02 |  | Cerebrovascular diseases | 84.54 | 1.80 | 2.18 |
| 4 | Injuries | 82.17 | 1.59 | 1.97 |  | Respiratory system diseases | 86.92 | 1.65 | 1.94 |  | Injuries | 84.27 | 1.53 | 1.85 |
| 5 | Heart diseases | 81.73 | 1.15 | 1.43 |  | Injuries | 86.78 | 1.51 | 1.77 |  | Heart diseases | 84.12 | 1.38 | 1.67 |
| 6 | Digestive system diseases | 80.89 | 0.31 | 0.38 |  | Endocrine, nutritional and metabolic diseases | 85.73 | 0.46 | 0.54 |  | Endocrine, nutritional and metabolic diseases | 83.09 | 0.35 | 0.42 |
| 7 | Nervous system diseases | 80.86 | 0.28 | 0.35 |  | Nervous system diseases | 85.63 | 0.36 | 0.42 |  | Nervous system diseases | 83.05 | 0.31 | 0.37 |
| 8 | Endocrine, nutritional and metabolic diseases | 80.85 | 0.27 | 0.34 |  | Digestive system diseases | 85.54 | 0.27 | 0.32 |  | Digestive system diseases | 83.04 | 0.30 | 0.36 |
| 9 | Infectious and parasitic diseases | 80.79 | 0.21 | 0.26 |  | Mental and behaviour disorders | 85.45 | 0.18 | 0.21 |  | Infectious and parasitic diseases | 82.93 | 0.19 | 0.23 |
| 10 | Genitourinary system diseases | 80.69 | 0.11 | 0.14 |  | Infectious and parasitic diseases | 85.41 | 0.14 | 0.16 |  | Mental and behaviour disorders | 82.87 | 0.13 | 0.16 |
